# Reversal of chemotherapy resistance in gastric cancer with traditional Chinese medicine as sensitizer: potential mechanism of action

**DOI:** 10.3389/fonc.2025.1524182

**Published:** 2025-02-20

**Authors:** Chencong Zhou, Kaihan Wu, Meng Gu, Yushang Yang, Jiatao Tu, Xuan Huang

**Affiliations:** Department of Gastroenterology, The First Affiliated Hospital of Zhejiang Chinese Medical University, Hangzhou, Zhejiang, China

**Keywords:** gastric cancer, chemoresistance, traditional Chinese medicine, sensitizer, signaling pathway

## Abstract

Gastric cancer (GC) remains one of the most common types of cancer, ranking fifth among cancer-related deaths worldwide. Chemotherapy is an effective treatment for advanced GC. However, the development of chemotherapy resistance, which involves the malfunction of several signaling pathways and is the consequence of numerous variables interacting, seriously affects patient treatment and leads to poor clinical outcomes. Therefore, in order to treat GC, it is imperative to find novel medications that will increase chemotherapy sensitivity and reverse chemotherapy resistance. Traditional Chinese medicine (TCM) has been extensively researched as an adjuvant medication in recent years. It has been shown to have anticancer benefits and to be crucial in enhancing chemotherapy sensitivity and reducing chemotherapy resistance. Given this, the mechanism of treatment resistance in GC is summed up in this work. The theoretical foundation for TCM as a sensitizer in adjuvant treatment of GC is established by introducing the primary signal pathways and possible targets implicated in improving chemotherapy sensitivity and reversing chemotherapy resistance of GC by TCM and active ingredients.

## 1 Introduction

According to global cancer statistics 2022, gastric cancer (GC) ranks fifth in terms of incidence and mortality, seriously threatening human health (1). Endoscopic resection is the main treatment for early-stage GC. Non-early operable GC is treated with surgery (2). Unfortunately, most patients are diagnosed with advanced unresectable or metastatic stages of disease at first, due to the lack of specific clinical symptoms.

In comparison to supportive therapy alone, combined chemotherapy can improve both survival rates and quality of life for patients with advanced or metastatic disease. A platinum fluoropyrimidine doublet has been the first-line therapy due to lower toxicity (3). Oxaliplatin is recommended for older patients because of its lower toxicity than cisplatin (4). First-line treatment based on irinotecan may be recommended as a first-line therapy option in patients with advanced or metastatic gastroesophageal cancer (3, 5, 6). For patients with Human Epidermal Growth Factor Receptor 2 (HER2) overexpression positive GC, Trastuzumab should be added to first-line chemotherapy [category 1 for cisplatin (7); category 2A for oxaliplatin] (3). Pembrolizumab can be added to this regimen to improve progression-free survival (8). When patients with HER2 overexpression are negative, the preferred regimen for Programmed cell death 1 ligand 1 (PD-L1) combined positive score (CPS)≥5 is nivolumab combined with fluorouracil and oxaliplatin (9). The selection of second-line and subsequent therapy is determined by performance status and the history of prior treatments. As a monotherapy or in combination with paclitaxel, ramucirumab is the preferred option for second-line and subsequent therapy (10, 11). As monotherapy or combination, docetaxel, paclitaxel, and irinotecan are recommended as a second-line therapy (12–14). The regimen of trifluridine and tipiracil is classified as category 1 recommendations for patients whose disease progressed after second-line chemotherapy (15). This treatment is suitable only for patients with low-volume GC, because of its strong cytotoxicity.

However, parts of GC cells escape cell toxicity and acquire stably resistant to drug during chemotherapy. The acquired resistance leads poor clinical efficacy and is the leading cause of chemotherapy failure in most patients. Currently, there are several strategies for overcoming chemoresistance in cancer, including discontinuous dosing, modifying drug concentrations, combination therapy, and the use of natural products (16). However, most of these strategies generally result in serious side effects, involve higher treatment costs, and technical difficulties (17). Natural products play an important role in treatment of diseases, especially for cancer and infection diseases. It has been reported that about half of the anti-cancer drugs approved by the Food Drug Administration (FDA) originate from either natural products or their derivatives (18). Traditional Chinese Medicine (TCM) has been extensively used clinically due to its strong specificity, high efficacy, and low toxicity. It not only inhibits tumor growth but also enhances chemotherapy efficacy and reverses chemoresistance when combined with traditional chemotherapy.

In this review, we aim to understand the mechanisms of chemotherapy resistance in GC and to explore the potential of TCM and its active components as chemotherapy sensitizers in reversing chemotherapy resistance and improving curative effect in GC. We hope TCM can be an innovative strategy to solve the difficulties of clinical anti-cancer treatments.

## 2 Mechanisms of chemoresistance for GC manuscript formatting

### 2.1 Drug efflux

**Figure 1:**
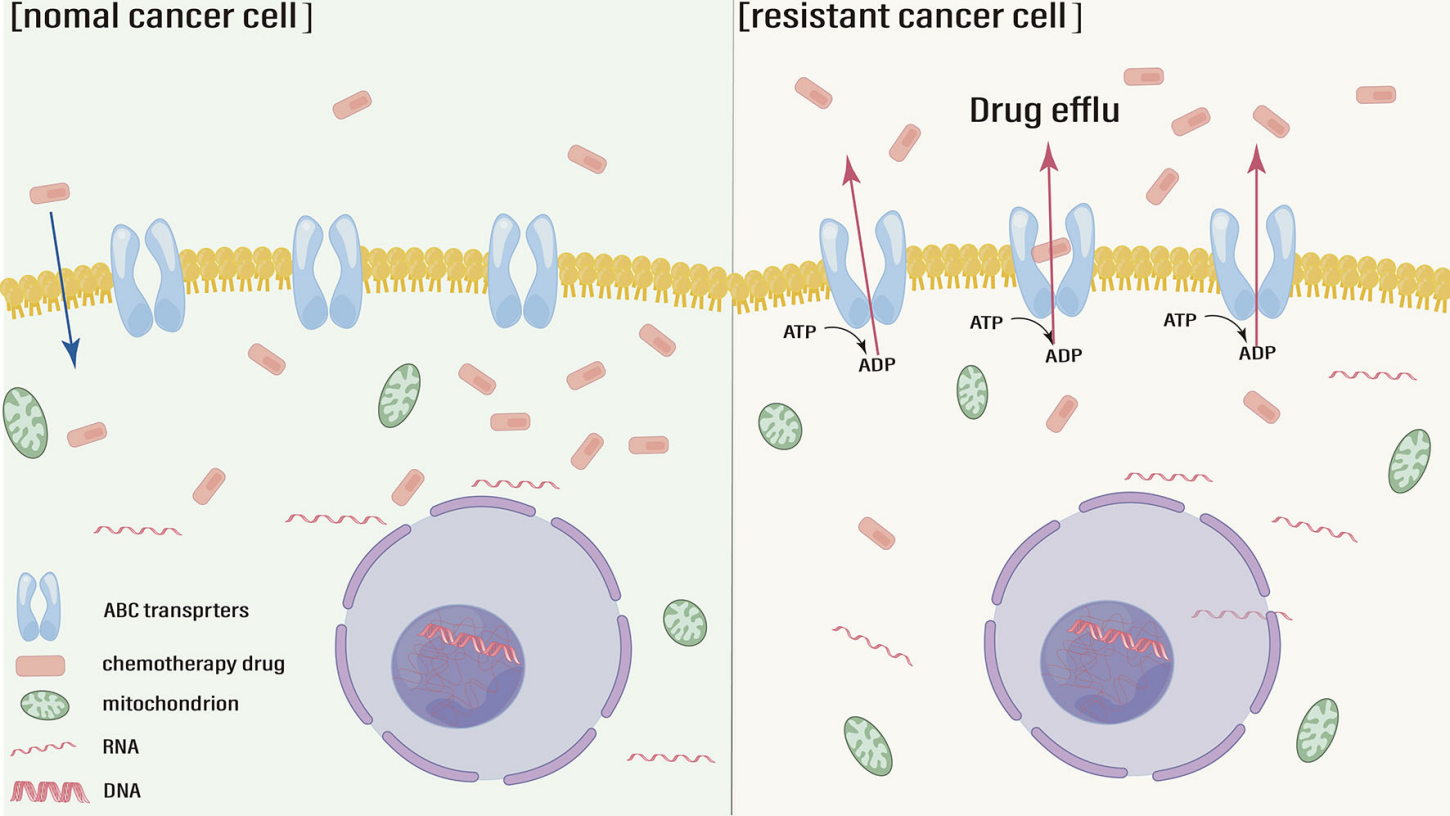
ABC transporters active drug efflux and chemotherapy resistance.

The intracellular concentration of antitumor drugs needs to remain within the effective concentration range to exert therapeutic effects. Compared with that in normal tumor cells, the intracellular drug concentration in resistant cells is well below the effective range due to enhanced drug efflux, reduced drug influx and drug sequestration (19). Chromodomain helicase DNA-binding protein 4 (CHD4) increases the cisplatin efflux and decrease the intracellular concentration, leading to drug resistance (20). Therefore, abnormally high expression of associated membrane proteins which mediate drug efflux is one of the causes leading to chemotherapy resistance (**Figure 1**). ATP-mediated ATP-binding cassette (ABC) transporter family is a major class of these membrane proteins. The level of the classical drug resistance-related protein P-glycoprotein (P-gp) gradually increases in gastric epithelial cells, GC cells and drug-resistant cells (21). PD-L1 promotes the expression of P-gp, by up-regulating the phosphatidylinositol-3-kinases (PI3K)/protein kinase B (Akt) signal pathway to enhance drug efflux and reduce the cell damage caused by cisplatin (22). One study found that a selective mammalian target of rapamycin complex 1/2 (mTOR1/2) dual inhibitor could enhance oxaliplatin-induced apoptosis by down-regulating the expression of p-gp (23). In addition, the function of ABC transporters, including P-gp, also relies on their subcellular location. A study showed that ABC transporters were more localized to the plasma membrane in SGC-7901 cells than in resistant cells (24). Multidrug resistance-associated protein 1 (MRP1) is another classic ABC transporters protein that is closely associated with the chemoresistance in GC. As a MRP1 regulator, the overexpression of Siva-1 could regulate the NF−κB pathway to GC cells escape certain chemotherapies (25). 

### 2.2 Inhibition of cell death

Programmed cell death can eliminate damaged cells or cells that replicate pathogens that are at risk of tumor transformation to maintain homeostasis in the body. These processes include apoptosis, ferroptosis, autophagy, pyroptosis, necrosis and necroptosis (26).

**Figure 2:**
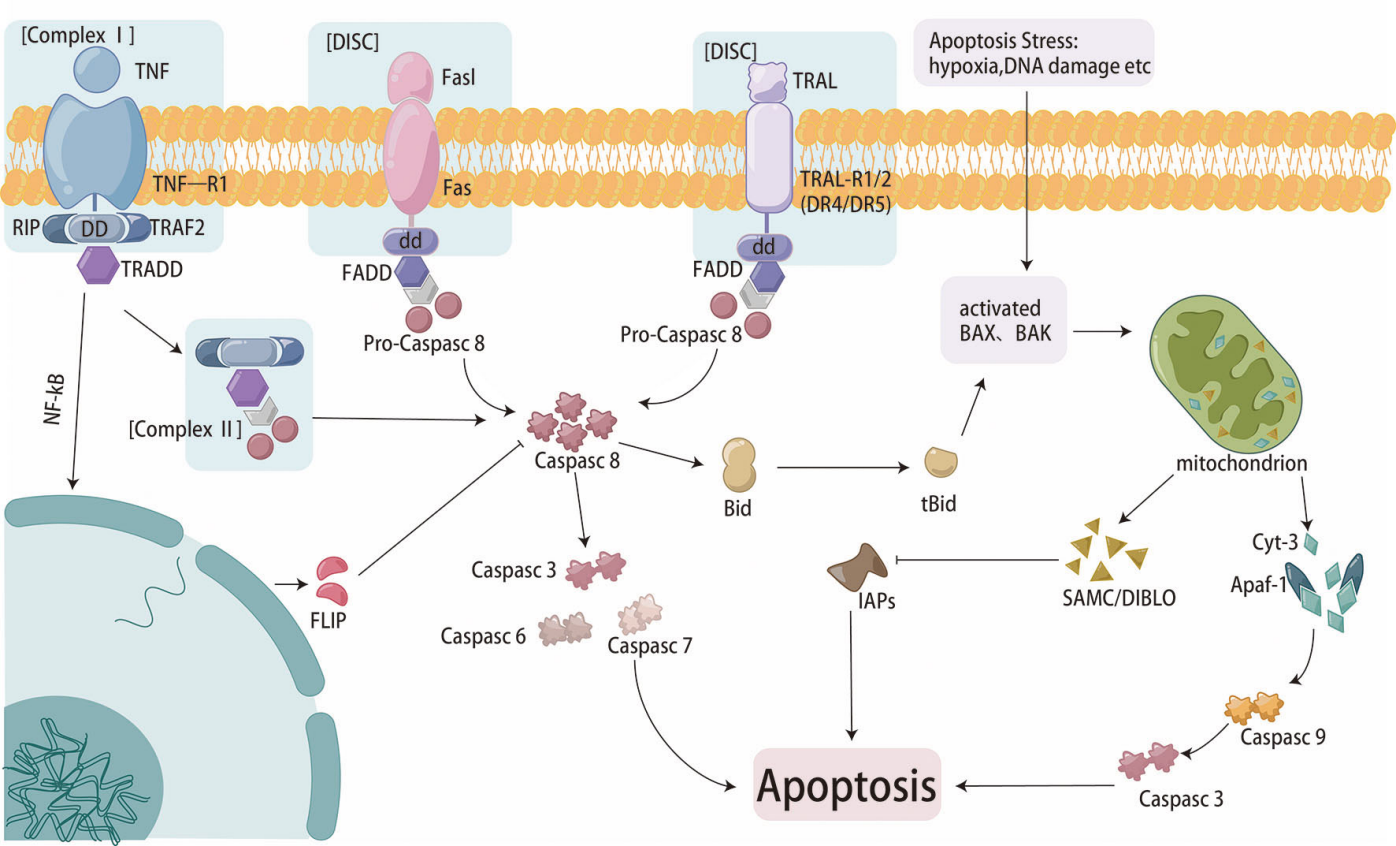
Cell apoptosis flowchart.

Apoptosis is essential for maintaining homeostasis. There are two pathways of apoptosis (**Figure 2**). One is that antiapoptotic and proapoptotic proteins interact with mitochondria to activate the mitochondrial pathway (19). The B-cell lymphoma-2 (Bcl-2) family is a pivotal protein in the mitochondrial pathway and regulates mitochondrial outer membrane permeabilization (MOMP). Paclitaxel can reduce the expression of Bcl-2 through direct binding, leading to apoptosis (27). BamH1 A fragment leftward reading frame 1 (BALF1) plays a role in maintaining Bcl-2 protein with anti-apoptotic characteristics stability, leading to cancer progression (28). The combined targeted therapy of pro-apoptotic Bax and anti-apoptotic Bcl-xl is a novel therapeutic strategy to overcome cancer progression and resistance (29). P53 is also a classical regulatory gene which can mediate the intrinsic apoptotic pathway (30). The pro-apoptotic protein p53 can be degraded through the exosome miR-769-5p-mediated ubiquitin-proteasome pathway, ultimately leading to cisplatin resistance (31). The other pathway is the extrinsic pathway, in which death receptors on the plasma membrane identify and bind special death ligands, inducing apoptosis (32). As a classical death ligand, tumor necrosis factor (TNF) binds to TNFR1 to recruit downstream molecules, leading to cell apoptotic (33). Studies have shown that TNF-α can regulate the nuclear factor kappa-beta (NF−κB) signaling pathway, driving cisplatin resistance (34).

**Figure 3:**
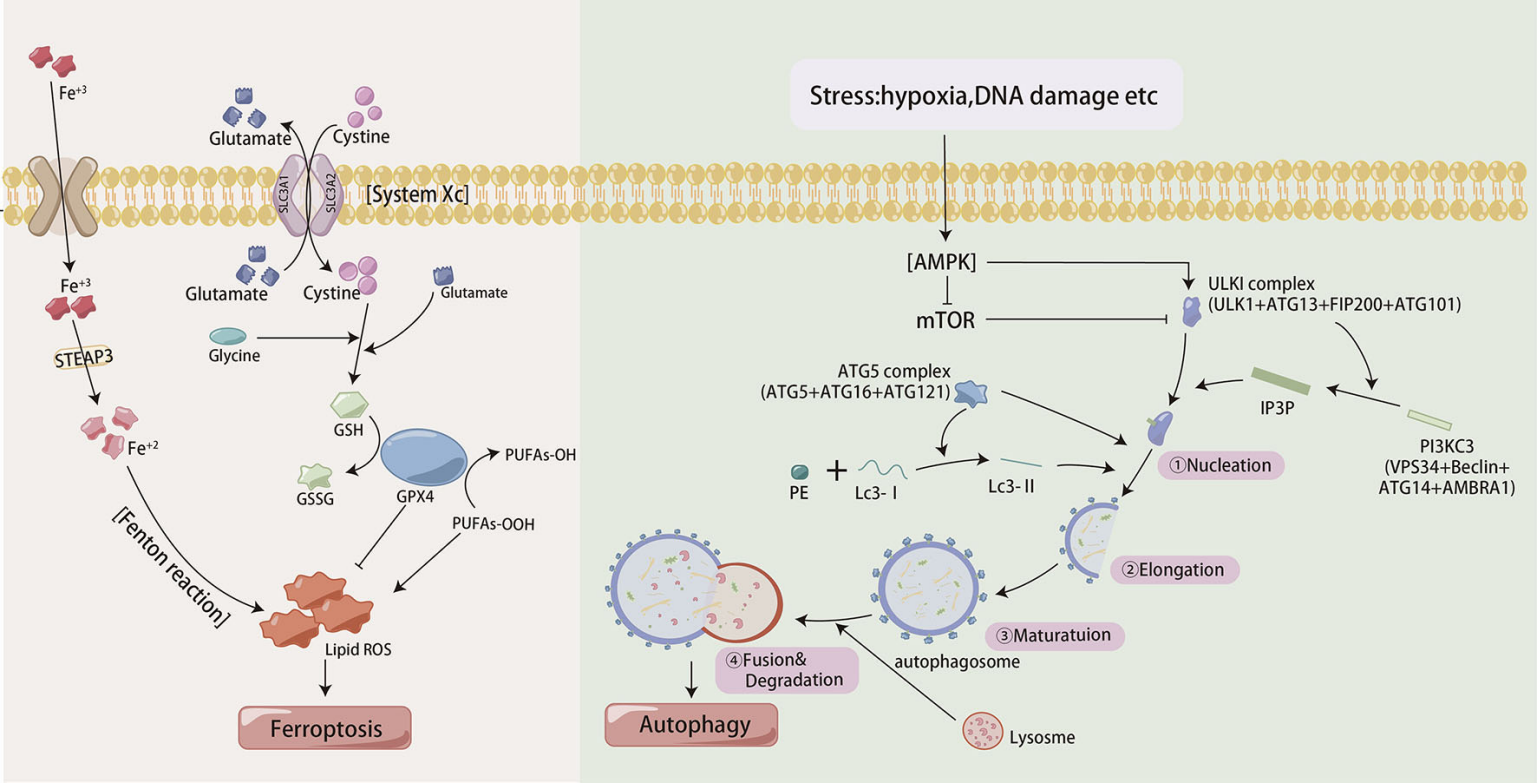
The process of autophagy and iron death in cells.

Autophagy, which is characterized by the self-degradation of intracellular components, plays dual roles in the resistance of GC to chemotherapy and relies on the intensity and duration of the stimuli (**Figure 3**). In the presence of persistent stimuli, autophagy, known as cytotoxic autophagy, has anticancer effects (35). However, autophagy, which is regulated by multiple proteins and signaling pathways, is also known as cytoprotective autophagy and can promote cancer cell resistance to chemotherapeutic agents (36, 37). Enhanced autophagy was activated by annexin A1 (ANXA1) via the PI3K/Akt pathway, resulting in oxaliplatin resistance in GC. Moreover, knockdown of ANXA1 could restore sensitivity to oxaliplatin (38). METase could inhibit autophagy through regulating the highly up-regulated in liver cancer (HULC)/Forkhead box protein M1 (FoxM1) pathway and enhance resistant cell sensitivity to cisplatin (39). Studies had shown that MFAP2 could promote autophagy and increase the resistance to cisplatin in GC, but the specific mechanism was not yet clear (40). so MEAP2 could be a potential therapeutic targe. As an essential deubiquitinase, USP13 maintained the stability of autophagy-related protein 5 (ATG5) to enhance autophagy and promoted imatinib resistance in cancer cells (41).

Ferroptosis is a unique iron-dependent mode of nonapoptotic regulated cell death, that involves iron-mediated accumulation of reactive oxygen species (ROS), oxidative stress and dysfunction of antioxidative defense (42) (**Figure 3**). Compared to GC cells, cisplatin-resistant GC cells exhibit lower levels of ferroptosis, evident by lower ROS, malondialdehyde (MDA) and lipid peroxidation and higher intracellular glutathione (GSH) levels (43). ATF3 blocked the Kelch-like ECH-associated protein 1 (Keap1)/NF-E2-related factor 2 (Nrf2) axis and induced ferroptosis, consequently restoring GC sensitivity to cisplatin. DNAJC12 had been shown to induce doxorubicin resistance through activating the Akt signal to repress cell ferroptosis (44). Cells subjected to continual chemotherapy often resist apoptosis but are sensitive to ferroptosis (45). So ferroptosis induction is considered a potential way to overcome chemoresistance.

### 2.3 Enhanced DNA damage repair

The DNA damage response (DDR) is a special repair system used to maintain genetic stability and integrity under stress conditions. Targeting DNA damage represents a primary mechanism employed by numerous chemotherapy agents (46). However, certain cancer cells can acquire drug-resistant phenotypes through the enhancement of DNA repair processes. Some nucleotide excision repair (NER) proteins are overexpressed in Pt-resistant cells. Spontaneous NER is one of the significant causes of platinum resistance. Poly (ADP-Ribose) polymerase 1 (PARP1), as an enzyme crucial for repairing DNA damage, can effectively repair damaged DNA by mediating abnormal activation of the base excision repair (BER) pathway, thereby resulting in oxaliplatin resistance (47). The excision repair cross-complementing gene (ERCC) is also a key gene involved in DNA repair. The overexpression of ERCC4 and ERCC3 may confer resistance to cisplatin by part of a mechanism involving the NER pathway (48). The high expression of ERCC1 is strongly associated with the risk of cisplatin in GC and is considered an independent predictor of the efficacy of platinum chemotherapy (49). The expression levels of ERCC1 and ERCC4 are inversely correlated with miR-138-5p in GC samples. Upon silencing miR-138-5p, the upregulation of ERCC1 and ERCC4 occurs, which subsequently reduces the sensitivity of GC cells to cisplatin (50).

### 2.4 Modulation of the tumor microenvironment

**Figure 4:**
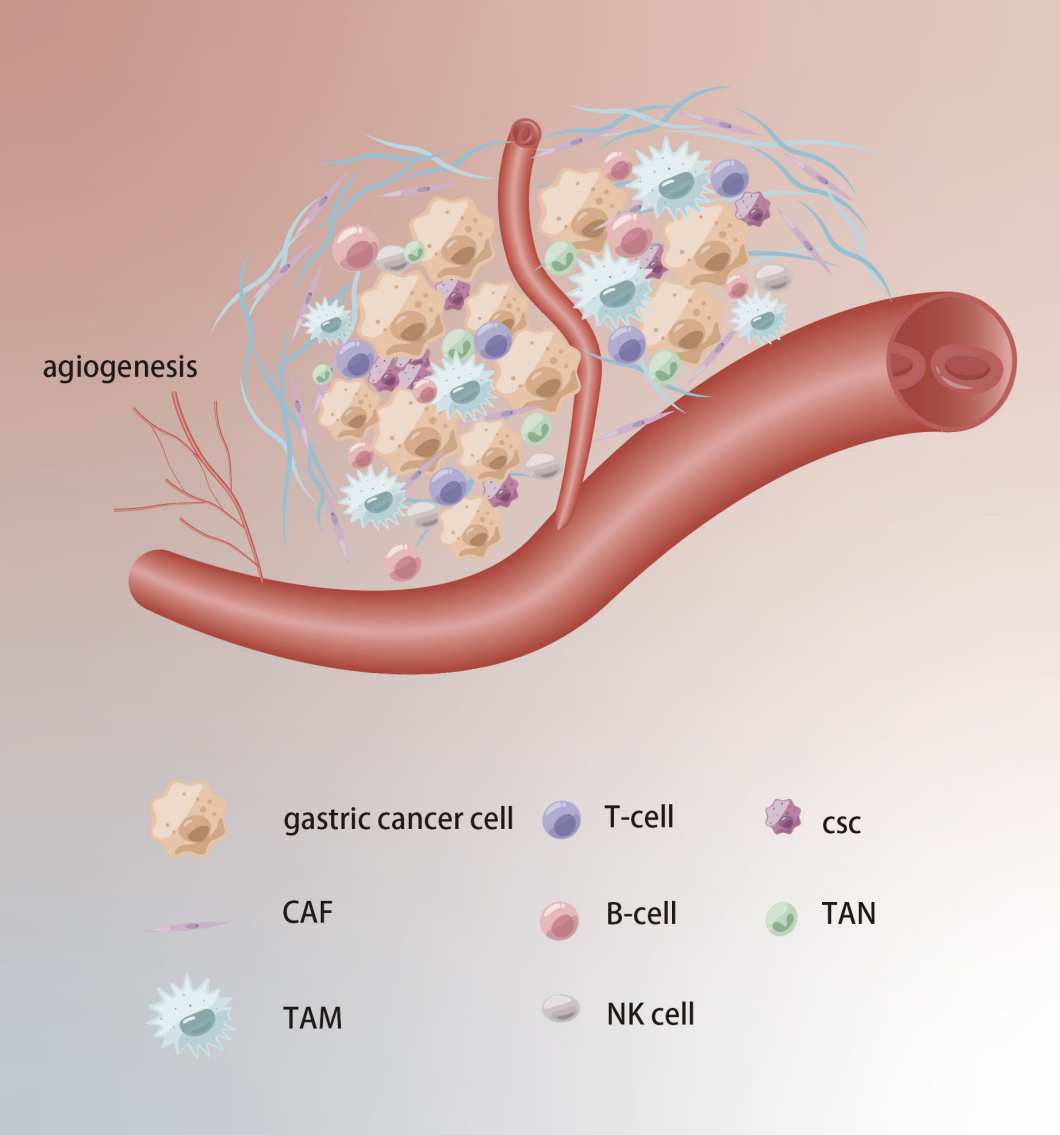
Tumor microenvironment in chemoresistance of GC. Extracellular matrix acts as a physical barrier to prevent drugs from entering. The TCM formation of new blood vessels, hypoxia and acidification contribute to chemoresistance.

The cellular environment in which tumors exist is referred to as TME, which comprises stromal cells, immune cells, and extracellular components and plays an essential role in tumor progression, drug resistance, and immune escape (**Figure 4**). Studies have found that the cancer-associated fibroblasts (CAFs) within the GC can secrete stromal cell-derived factor-1 (SDF-1) by activating the Hippo pathway, thereby inducing resistance to 5-Fu (51). Tumor-associated macrophages (TAMs) with the M2 phenotype are also one of the main causes of drug resistance. CXCL5 derived from M2-TAMs induces 5-Fu resistance by regulating the PI3K/Akt/mTOR pathway (52). It’s widely believed that mesenchymal stem cells (MSCs) can mediate the PD-L1, thus leading to GC invasion, metastasis and therapy escape. Wang et al. found MSCs could enhance cisplatin resistance of GC cells exposed to cisplatin through regulating PD-L1 to promote the expression of multi-drug resistance 1 (MDR1) and Reds1 (53). For noncellular components, approximately 50–60% of locally advanced solid tumors show areas of hypoxia. As an important gene of hypoxia, the high expression of HIF-1α can mediate cellular resistance to cisplatin and paclitaxel (54, 55). Regarding trace elements, the levels of zinc, and manganese in GC tissues are markedly higher compared to those in adjacent normal tissues (56).

### 2.5 Intensification of epithelial-mesenchymal transition

EMT refers to the process of phenotypic transformation of epithelial-like cells into mesenchymal cells. After EMT, cancer cells lose epithelial characteristics and acquire higher migration and transfer ability (57). EMT is involved in several signaling pathways which include the Notch, Wnt, and TGF-β signaling pathways (58). At the molecular level, the changes in a variety of cell adhesion molecules, such as E-cadherin and N-cadherin, indicate the occurrence of EMT. Resistant cells are apt to metasize. The expression level of Ras-related protein 31 (Rab31) is negatively correlated with the sensitivity of cisplatin to stomach adenocarcinoma, and it can activate Twist1 through regulating the Stats/Mucin 1 (MUC-1) signaling, thereby mediating cisplatin resistance and metastasis (59). Liu et al. pointed out that the overexpression of Wilms tumor 1 associated protein (WTAP) was significantly correlated with poor cancer prognosis, as it facilitated the EMT in GC cells by modulating TGF-β expression and stability of mRNA, consequently leading to multiple chemotherapy resistance and metastasis (60). Adenosine deaminases acting on RNA1 (ADAR1) has been shown to be involved in occurrence and development of GC, and it regulates the protein expression levels of EMT-related markers via the antizyme inhibitor1 (AZIN1) pathway. Knockout of ADAR1 can inhibit the metastatic, as well as enhance sensitivity to cisplatin (61).

### 2.6 Cancer stem cell

**Figure 5:**
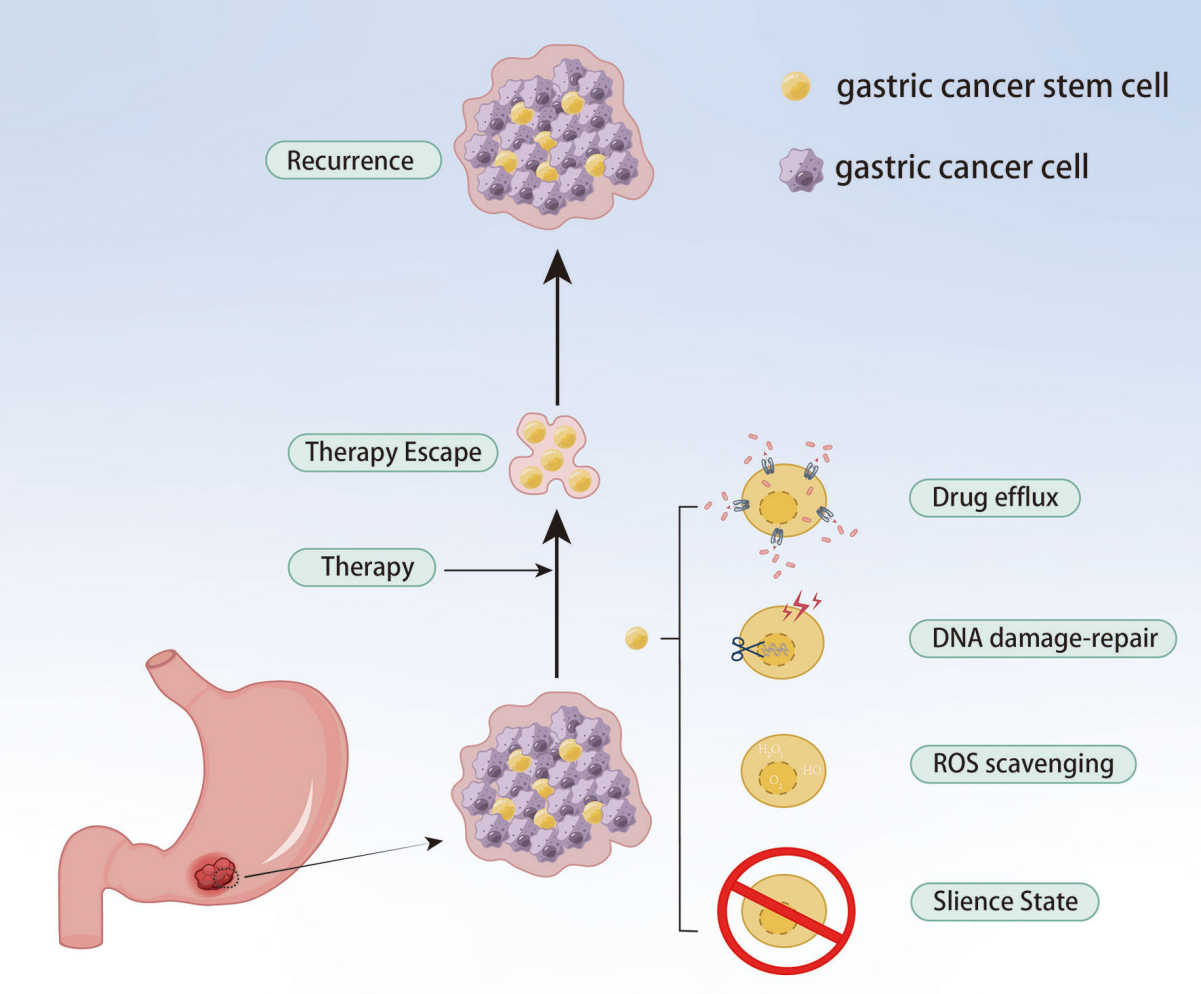
gCSCs keep their sustained growth, metastasis and gain chemoresistance by escaping chemical therapy.

Although only a small proportion of GC cells, GC stem cells (gCSCs) are considered the key contributor to tumor initiation, metastasis, recurrence and treatment failure. gCSCs can develop drug resistance by affecting drug efflux, apoptosis, DNA damage repair, TME and EMT, as well as proliferate after escaping chemotherapy, eventually resulting in tumor recurrence and metastasis (62) (**Figure 5**). As a highly expressed gene in resistant cells, PRKA kinase anchor protein 8L (AKAP-L8) can promote GC cells to acquire stem cell-like features by maintaining the stability of Stearoyl-CoA desaturase 1 (SCD1) via an IGF2 mRNA binding protein 1 (IGF2BP1)-dependent manner, resulting in resistance to oxaliplatin (63). Ukai et al. also found that KH domain-containing RNA-binding signal transduction-associated protein 3 (KHDRBS3) might play a role in stem cell-like characteristics by mediating CD44 variant expression, thereby reducing the sensitivity of GC cells to 5-Fu (64). Metallothionein 1 M (MT1M) plays a key role in tumor progression and formation, and its expression is positively correlated with clinical prognosis. The overexpression of MT1M can inhibit stem cell characteristics and reverse 5-Fu resistance by targeting Glioma-associated oncogene homolog 1 (GLI1) and affecting GLI1 ubiquitination (65). Wnt1 has been proven to be a potential therapeutic target. Tan et al. found the Wnt1-SOX4 positive feedback loop could maintain gCSCs self-renewal and tumorigenicity and associate with the resistance of 5-Fu and oxaliplatin (66).

### 2.7 Metabolic reprogramming

Metabolic reprogramming of cancer cells can efficiently obtain and utilize nutrients to adapt to various signals of TME and facilitate survival, proliferation and drug resistance. Therefore, it may be a foundation for the development of drug resistance. Cancer cells preferentially produce energy through glycolysis rather than oxidative phosphorylation, the phenomenon referred to as aerobic glycolysis. Xu et al. found that Far upstream element-binding protein 1 (FUBP1), which is involved in regulation of target gene transcriptional *in vivo*, could positively correlate with aerobic glycolysis and induce oxaliplatin resistance by regulating glycolysis in GC cells (67). He et al. pointed out that pyrimidine biosynthesis could accelerate glycolysis via activating Notch signaling and enhancing the expression of c-Myc, leading to hindering the efficacy of chemotherapy (68). Maintenance complex component 10 (MCM10) is also observed to be enriched in the glycolysis-related pathway, leading to an enhancement of stemness characteristics in GC cells and contributing to paclitaxel resistance (69). Glucose-regulated protein 75 (GRP75) is highly expressed in cisplatin-resistant GC cells. Knockdown of GRP75 can alter the metabolic reprogramming through blocking anti-oxidation/apoptosis-related progress, thereby enhancing the sensitivity to cisplatin (70).

### 2.8 Non-coding RNAs and exosomes

Non-coding RNAs, involved in miRNAs, lncRNAs, and circRNAs, and exosomes have been extensively studied for their roles in the chemoresistance of GC. Circ_0006089 is highly expressed in GC-resistant cells, while it can induce GC cells resistant to oxaliplatin through mediating Neuropilin 1 (NRP1) expression via sponging miR-217 (71). Fei et al. pointed out that circ_0008315 could accelerate GC progression and hinder therapeutic efficacy of cisplatin by enhancing GC cell stemness property (72). Overexpression of miR-30c-5p can directly target the 3’UTR of Lactate Dehydrogenase A (LDHA) to block glycolysis, thereby reversing resistance (73). The levels of regulator of reprogramming (ROR) and high mobility group protein. A2 (HMGA2) are significantly upregulated, but miR-519d-3p is downregulated in GC tissues and cells. Knocking down ROR can restrain cisplatin resistance in GC cells through targeting miR-519d-3p (74). Chen et al. found that the exosomal circ-0091741 can induce apoptosis and oxaliplatin resistance through blocking miR-330-3p combination with tripartite motif 14 (TRIM14) and activating the Wnt/β-catenin signaling via stabilizing dishevelled 2 (Dvl2) (75).

## 3 TCM in the treatment of GC

It is well known that active ingredients extracted from Chinese herbs have therapeutic effects on GC. Artemisinin, which is the active ingredient extracted from the leaves and roots of Artemisia annua, is frequently used to cure malaria. In recent years, artemisinin and its derivatives have been shown in numerous investigations to have anticancer properties (76). In both cellular and mouse models, the derivative dihydroartemisinin successfully suppressed tumor progression and vasculogenic mimicry formation (77). Moreover, the combination of dihydroartemisinin and anlotinib can increase the rate of apoptosis and prevent angiogenesis, migration, and invasion of cells (78). Li et al. have discovered that after dihydroartemisinin treatment, E-cadherin showed high expression, while vimentin, Akt, p-Akt, and Snail showed low expression in SGC7901 cells, thereby effectively blocking EMT and inducing cell apoptosis (79). As another derivative, artesunate has been shown to induce apoptosis by downregulating Cox-2 expression and impeding mitochondrial function (80). Su et al. have found that artesunate, dihydroartemisinin, and artemisinin could effectively reduce the incidence of GC in mouse models and prevent the occurrence of Helicobacter pylori-induced GC (81).

As a natural flavonoid, quercetin is the primary active ingredient in Chinese herbs such as hawthorn, licorice, and knotweed, and it has anticancer effects. Through network pharmacology, quercetin is involved in regulating apoptosis, proliferation, metabolism, and oxidative stress of GC and treating GC through PI3K/Akt signaling, EGFR tyrosine kinase inhibitor resistance, Rap1 signaling, ErbB signaling, FoxO signaling, and Ras signaling pathways (82). According to Shen et al., quercetin could inhibit the progression of GC by blocking the PI3K/Akt pathway and inducing mitochondria-dependent apoptosis (83). Ding et al. have discovered that through targeted inhibition of SLC1A5 expression, quercetin could also hasten iron precipitation in GC cells, leading to ferroptosis (84). Additionally, quercetin can upregulate pyroptosis-related genes (GSDMD, GSDME, cleaved CASP1, NLRP3) and activate the pyroptosis pathway to suppress cell proliferation (85).

A variety of natural active ingredients derived from the Chinese herb Salvia miltiorrhiza play an important role in the adjuvant treatment of GC. Tanshinone II can induce ferroptosis and inhibit cell proliferation in BGC803 and NCI-H87 cells by increasing lipid peroxidation and upregulating the expression of ferroptosis markers Ptgs2 and Chac1 (86). Huo et al. also found that tanshinone II could promote apoptosis (87). According to Xiao et al., tanshinone I could effectively induce cell ferroptosis (88). Moreover, tanshinone I can reduce inflammation and inhibit precancerous lesions of GC by reversing abnormal expression of E-cadherin and N-cadherin (89). Another ingredient, diterpenoid tanshinones, has been proven to regulate the expression of angiogenic factors and inhibit tumor angiogenesis (90). Wang et al. have found that a neutral polysaccharide fraction (SMPA) prepared from the roots of Salvia miltiorrhiza could be used as a potential immunomodulator. It improved the TME, stimulated splenocyte proliferation, promoted anti-inflammatory cytokine production, and augmented the killing activity of natural killer cells and cytotoxic T lymphocytes in GC rats (91).

Astragalus IV is one of the active ingredients of Astragalus, which has anti-inflammatory, hypoglycemic, antifibrotic, and anticancer activities (92). Zhu et al. discovered that Astragalus IV could dramatically lower GC cell invasion and migration through reversing TGF-b1-induced EMT (93). Astragalus IV has also been shown to be able to reshape TME and correct CAFs dysfunction caused by dysregulation of mic RNA expression, thereby inhibiting GC cell proliferation, invasion, and migration (94). For precancerous lesions of gastric cancer, Astragalus IV provides therapeutic effects. Zhang et al. discovered that the PLGC rats’ stomach epithelial dysplasia area decreased and their epithelial cells became more symmetrical after Astragalus IV therapy (95). Astragalus saponins can inhibit angiogenesis. In GC cells treated with Astragalus saponins, VEGF, MMP-9, and MMP-3 levels decreased significantly, and the cells stopped in the G2/M stage, thus inhibiting tumor development and invasion (96). Calycosin, as another active ingredient of Astragalus, promotes apoptosis through mediating ROS, thus playing an anticancer role (97). In addition, Li et al. found that calycosin could also improve IM, dysplasia, and protect the stomach in MNNG-induced PLGC rats (98).

Curcumin is a polyphenolic compound derived from turmeric, which has broad-spectrum anticancer effects. The activity and migration of GC cells treated with curcumin decreased in a concentration-dependent manner, which may be related to down-regulating the expression of related genes in the PI3K pathway (99). Curcumin can also inhibit GC cell proliferation by activating P53 and induce apoptosis and autophagy (100).

## 4 Potential targets and mechanisms of action

### 4.1 PI3K/Akt signaling pathway

**Figure 6:**
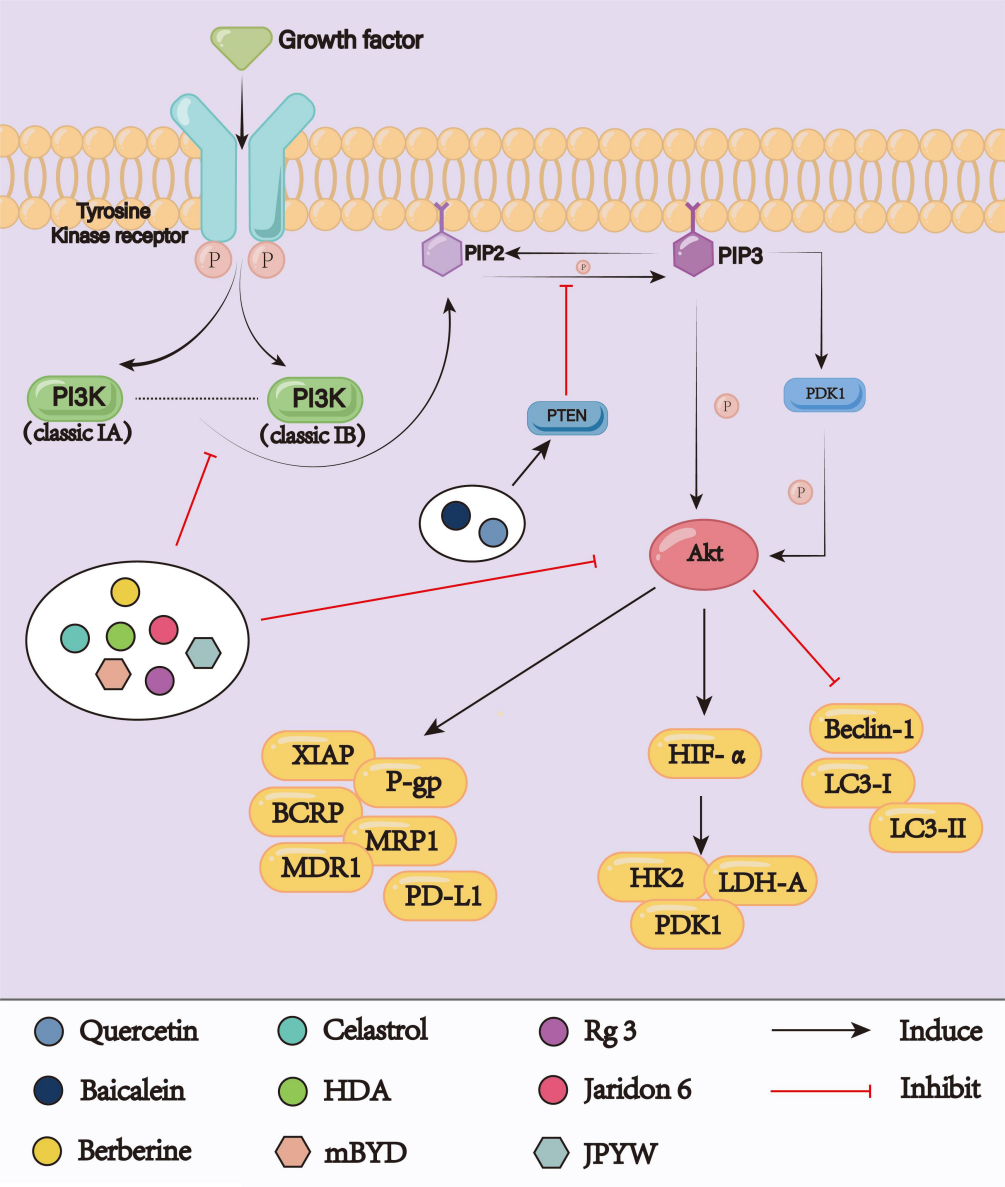
the PI3K/Akt signaling pathway.

The PI3K/Akt signaling pathway is one of the vital intracellular signaling pathways (**Figure 6**). PI3K, as a classic lipid kinase, participates in various cellular functions, including growth, proliferation, differentiation, and survival. PI3K can be activated, turning into PI3K-phosphorylated phosphatidylinositol 3,4,5-trisphosphate (PIP3), when stimulated by extracellular signals, such as EGFR, PDGF, RGF, and IGF, thereby promoting signal transduction cascades (101, 102). Akt, which is the most important downstream target, directly responses to PIP3, resulting in regulating downstream effectors (103). The PI3K/Akt signaling pathway is considered a significant cause of chemoresistance in cancer therapy. By controlling key apoptosis factors, including XIAP and the Bcl-2 family, the PI3K/Akt pathway prevents apoptosis and eventually results in chemoresistance. According to numerous studies, overactivation of Akt stimulates Bcl-2 while inhibiting Bax, thereby promoting cancer cell survival (104). Liu et al. demonstrated that overactivation of the PI3K/Akt signaling pathway upregulated the expression of Bcl-2 in cancer cells and significantly inhibited cisplatin-induced apoptosis (105). As a primary apoptosis inhibitor, XIAP can bind to caspase-9 and caspase-3 to block active caspase and inhibit apoptosis. In parts of cancer cells, XIAP is highly expressed, which is thought to be related to drug resistance. XIAP, which is downstream of Akt, upregulates the PI3K/Akt cell survival signaling pathway to prevent apoptosis (106). In addition, abnormal activation of the PI3K/Akt pathway mediates the expression of ABC transporters, which increases drug efflux and reduces drug response through up-regulation of P-gp, BCRP, and MRP1 expressions, thus leading to chemoresistance (107). The metabolic reprogramming of cancer cells to increase energy supply during chemotherapy is one of the causes of drug resistance. As an important regulator of glucose metabolism, Dong et al. found that through ROS-mediated activation of the PI3K/Akt signaling pathway, HIF-1α was up-regulated in cancer cells, inducing glucose metabolic reprogramming, and eventually cancer cells acquire resistance to anti-tumor drug (108). Consequently, targeting the PI3K/Akt signaling pathway may play a pivotal role in overcoming chemoresistance.

Research had demonstrated that quercetin may successfully cause chemosensitization and reverse MDR. Through network pharmacology and molecular docking studies, Guo et al. demonstrated that the reversal of MDR by quercetin was closely associated with the PI3K/Akt signaling pathway (109). Following additional investigation, it was found that the expression of phosphatase and tensin homolog deleted on chromosome 10 (PTEN) is concentration-dependently upregulated in quercetin-treated KATOIII/OXA cell lines, which blocked the phosphorylation of the PI3K/Akt signaling pathway, limited P-gp activity, and increased the intracellular level of oxaliplatin in KATOIII/OXA cells, thereby reversing oxaliplatin resistance (110). As an active component of celastrus and triperygium, celastrol exhibits significant broad-spectrum anticancer activities for the treatment of various cancers. Zhan et al. investigated the impact of celastrol on the PI3K/Akt signaling pathway and the expression levels of related proteins in the SGC7901/DDP cell line. Their findings indicated a significant reduction in the expressions of p-PI3K, p-mTOR, and p-4EBP1 in the SGC7901/DDP cells treated with celastrol, leading to PI3K/Akt signaling pathway inhibition. Additionally, the combination of celastrol and cisplatin reduced the expression of P-gp and MRP1 in the SGC7901/DPP cells. Therefore, celastrol was shown to reverse cisplatin resistance by inhibiting PI3K/Akt signaling and downregulating drug resistance-related protein expression (111). Zhang et al. had demonstrated that dihydroartemisinin exhibited anti-cancer efficacy in the treatment of SGC7901/DDP cells, and significantly enhanced the levels of autophagy-related proteins such as Beclin-1 and LC3II by inhibiting the PI3K/Akt/mTOR signaling pathway while concurrently downregulated P-gp, thereby increasing sensitivity to cisplatin (112). Ginsenoside Rg3 could both sensitize GC cells to cisplatin-induced cell death and relieve miR-420-mediated cisplatin resistance. The fundamental process might entail that Rg3 enforced SOX2 expression and exerted cytotoxic effects due to the downregulation of SOX2 in AGS/DDP GC cells, resulting in inhibiting downstream PI3K/Akt signaling pathway hyperactivation (113). Jaridon 6, a diterpene derived from Rabdosia rubescens, is thought to have the ability to activate autophagy. According to research by Fu et al., Jaridon 6 could effectively inhibit the proliferation of paclitaxel-resistant cells and 5-Fu-resistant cells both *in vivo* and *in vitro*. This may reduce the activity of the sirtuin1 (SIRT1) enzyme via the PI3K/Akt signaling pathway (114). Chen et al. discovered that baicalein could increase the susceptibility of stomach cancer cells to 5-Fu under hypoxic conditions. According to the mechanism study, baicalein could downregulate downstream important glycolytic enzymes (HK2, LDH-A, PDK1) by encouraging the accumulation of intracellular PTEN and inhibiting the activation of the PI3K/Akt signaling, thereby reversing the hypoxia-induced 5-Fu resistance (115). The BGC823 and SGC7901 cell lines treated with berberine had markedly reduced MDR1 and MRP1 expressions. In the meantime, cisplatin resistance was reversed by berberine and cisplatin combination therapy, which suppresses the PI3K/Akt signaling and increases caspase-3 and caspase-9 activation to cause apoptosis (116).

A traditional prescription that demonstrated an enhanced immune response through anti-oxidation and anti-inflammation is the decoction of Buzhong Yiqi (BZYQ). To prevent GC growth and immunological escape, Liu et al. had used modified-BZYQ (mBZYQ), guided by clinical practice and traditional Chinese medicine theory. The levels of PI3K, p-PI3K, and p-AKT in BGC823 cells were decreased after the intervention of mBZYQ, which resulted in activation of T lymphocytes and inhibited the PD-L1 expression in GC. This worked in concert with 5-Fu to prevent the progression of GC (117). The decoction of Jianpi Yangwei (JPYW) could increase the apoptosis of BGC825/5-Fu cells by lowering the p-Akt to Akt ratio, which inhibited the expression of MDR1 and MRP1 while increasing Bcl-2 and caspace-3. Combining JPYW with the pathway inhibitor LY294002 could further reduce MDR1 expression and encourage apoptosis (118).

### 4.2 NF−κB signaling pathway

**Figure 7:**
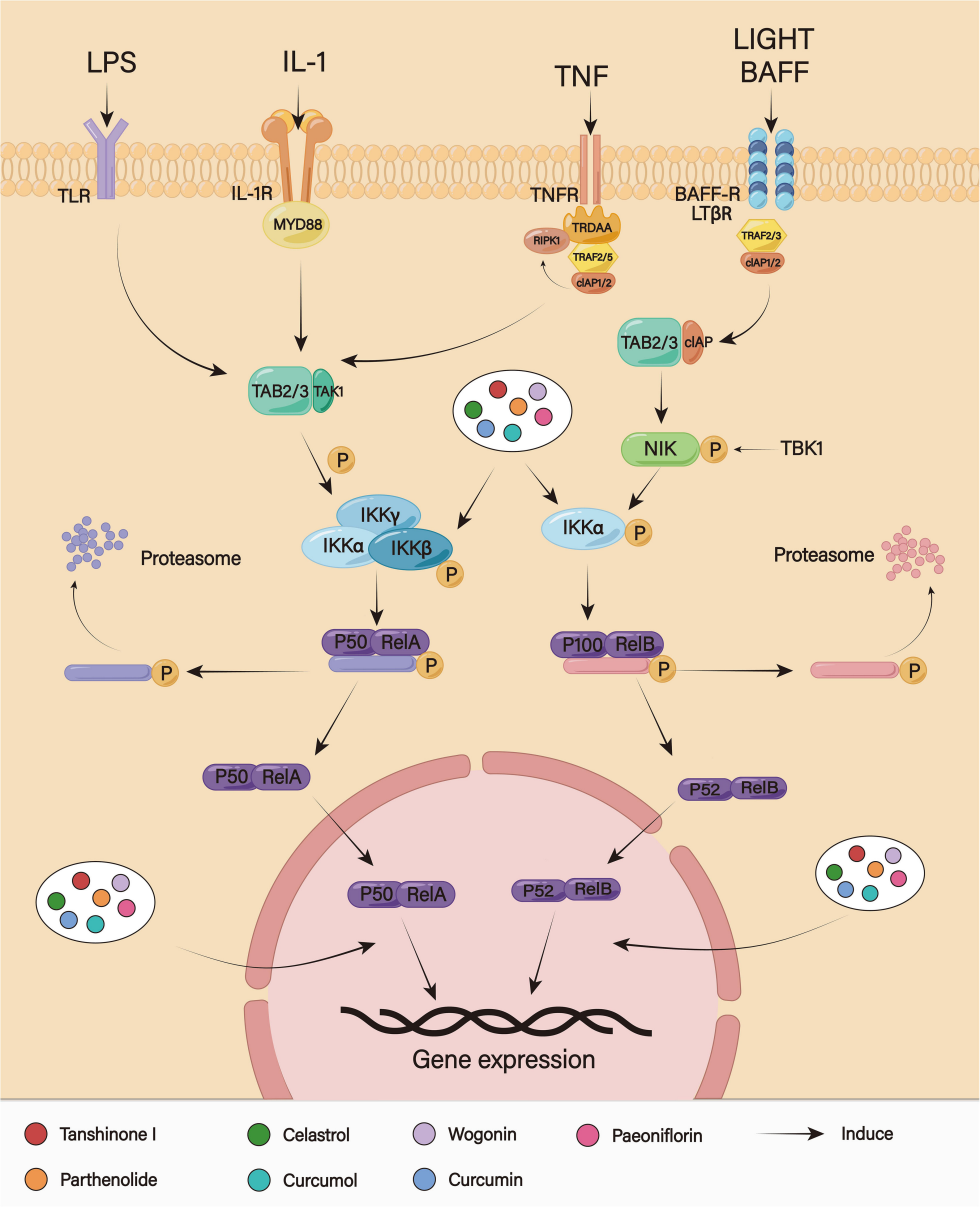
The NF−κB signaling pathway.

The transcription factor a common gene regulator, NF−κB plays a role in the recoding of cell adhesion molecules, cytokines, and cytokine receptors (119). The occurrence and development of tumors, including their proliferation, differentiation, migration, and resistance to radiation and chemotherapy, are intimately linked to aberrant NF−κB activation. The NF−κB family’s REL homology domain regulates DNA binding, dimerization, and interactions with inhibitory factors called IκB proteins (120). NF−κB complexes are found in the cytoplasm of the majority of untransformed cell types. They also prevent nuclear uptake and DNA binding, which results in transcriptional inactivation (121). The IKK complex is made up of the catalytic subunits IKKα and IKKβ as well as the regulatory component IKKγ (NEMO) (122). Following the activation of their respective receptors by signaling molecules, the IKK complex activates and causes the ubiquitin proteasome pathway to hydrolyze NF−κB complexes. This leads to the release of NF−κB dimers from cytoplasmic inhibition and their translocation to the nucleus, which in turn drives transcription of the target gene (123). The NF-kB signaling pathway is also a driving factor for chemoresistance in parts of malignant tumors (**Figure 7**). After stimulating, the NF-kB pathway may participate in the regulation of P-gp expression through binding to the MDR1 gene promoter region. According to Song et al., Adriamycin-resistant cells exhibited a marked overexpression of P-gp and NF-kB signaling pathway, and NF-kB inhibitor BAY1-7082 could overcome drug resistance through blocking the pathway and downregulating P-gp expression (124). Aronia berry extracts had also been demonstrated to reverse gemcitabine resistance, through inhibiting the NF-kB pathway in Pancreatic ductal adenocarcinoma (PDAC) cells to target the expressions of MYD88 and P-gp (125). Moreover, it has also been demonstrated that NF-kB pathway is associated with apoptosis, causing the activation of genes linked to anti-apoptosis through target genes, which results in apoptosis escape and reducing drug efficacy. According to one study, MIR55HG could mediate cisplatin and 5-Fu resistance of GC by triggering the NF-kB signaling pathway and preventing cisplatin and 5-Fu-induced apoptosis (126). Yan et al. found that IL-33 could activate the NF-kB signaling pathway, which in turn could downregulate caspase-3 expression, while increase the expressions of Bcl-2 and Bax, leading to reduce the sensitivity of acute myeloid leukemia to chemotherapy (127). The NF-kB pathway also participates in TME-related chemoresistance. CAFs can mediate platinum resistance in GC and PDAC by producing IL-8 and activating the NF-kB pathway (128, 129). It has been demonstrated that the occurrence of EMT is closely related to the NF-κB signaling pathway. Fu et al. found that the NF−κB signaling pathway contributes to cisplatin resistance in GC by promoting CD133-induced EMT (130).

Tanshinone I had been shown to inhibit cervical cancer’s growth and resistance to chemotherapy in a way that was dependent on kirsten rat sarcoma virus oncogene homologue (KRAS) (131). Tanshinone I had been shown by Wang et al. to prevent resistant GC cells from proliferating and spreading. The levels of phospho-IKK-a/b, NF−κB, GSDME-NT, cleaved caspase-8, and cleaved caspase-3 were significantly elevated in BGC823/DDP and SGC7901/DDP cells treated with tanshinone I. This implied that tanshinone I reversed cisplatin resistance by triggering pyroptosis through the signaling pathways of NF-kB and caspase -3/-8. In contrast, when combined with tanshinone I, cisplatin’s anti-tumor activity was enhanced and the growth of GC tumors transplanted subcutaneously in mice was inhibited (132). By inhibiting NF-kB-related genes, parthenolide could increase chemosensitivity to paclitaxel by suppressing NF-κB phosphorylation. In comparison to the group treated with paclitaxel alone, the combination treatment of parthenolide and paclitaxel dramatically inhibited proliferation and promoted apoptosis in MKN45 GC cells. Additionally, in all three GC cell lines, parthenolide tended to have a synergistic antitumor impact when combined with paclitaxel and cisplatin (133). Combined treatment of 5-Fu with celastrol effectively inhibited proliferation and induced apoptosis, which may be related to reducing the expression of IκB kinase and NF-κB, inhibiting the NF-κB P60 subunit, and inhibiting the signaling pathway. 5-Fu at lower concentrations could still exert higher anti-cancer effects; at the same time, the adverse reactions caused by cytotoxicity could be alleviated (134). GC cells’ sensitivity to cisplatin had purportedly been connected to curcumol, a bioactive sesquiterpenoid that was isolated from several plants in the genus curcuma (135). According to Hu et al., curcumol could inhibit the NF-κB pathway, and curcumin-treated GC cells exhibited a large rise in miR-7, which improved the sensitivity of GC to cisplatin. However, downregulation of miR-7 or miR-7 knockdown led to increased NF-κB p65 (RELA) and SNAIL protein levels in GC cells, thereby blocking the sensitizing effects of curcumol (136). Wogonin is a flavonoid compound found in Scutellaria baicalensis Georgi (Huang Qin). According to Zhao et al., wogonin made the human GC cells MGC803 more susceptible to 5-Fu-induced apoptosis. Possible mechanisms included suppression of NF-kB nuclear translocation and I-kB phosphorylation and dihydropyrimidine dehydrogenase (DPD) downregulation to slow down drug metabolism, which boosted the anti-tumor efficacy of low dose 5-Fu in MGC803 cells (137). Curcumin further reduced NF-kB activation and downregulated the expression of downstream anti-apoptotic gene products, including Bcl-2 and Bcl-xl, in the SGC7901 cell line treated with chemotherapeutics (etoposide and doxorubicin). This suggested that curcumin may promote apoptosis and reverse chemoresistance through the NF-kB signaling pathway (138). Wu et al. demonstrated that NF-kB activity in the nuclei of SGC7901 cells was significantly inhibited following treatment with aeoniflorin, indicating that paeoniflorin may promote 5-Fu-induced apoptosis by preventing IkBα phosphorylation and reducing NF-kB nuclear translocation (139).

### 4.3 Mitogen-activated protein kinases signaling pathway

**Figure 8:**
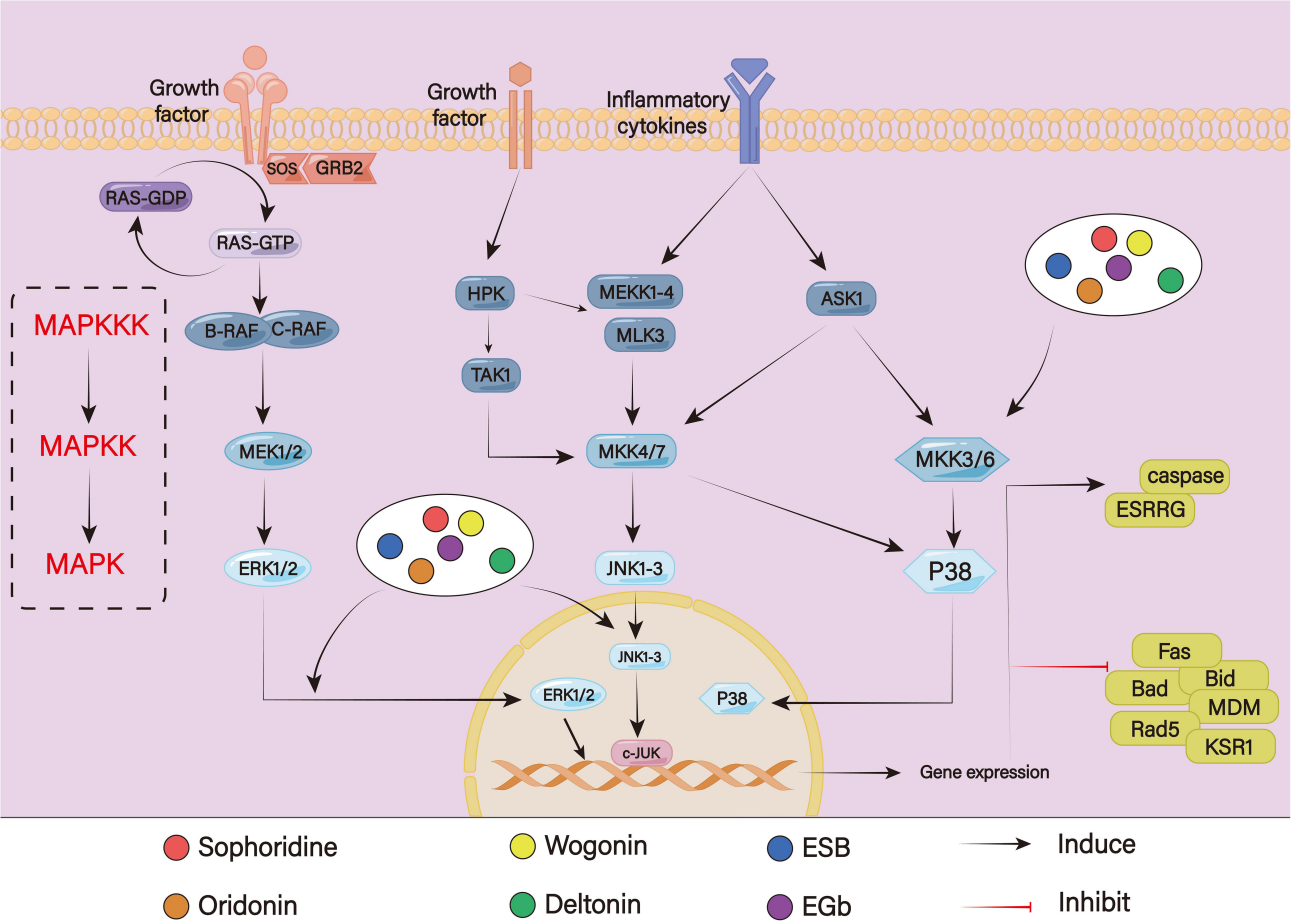
The MAPK signaling pathway.

Protein kinases in the MAPK pathway are continuously activated to transmit a variety of input signals, such as hormones, cytokines, cell growth factors, endogenous stressors, and environmental stimuli (140). This sets off a series of events that support several biological processes. The four cascades that make up the MAPK pathway are extracellular signal-regulated kinase (ERK) 1/2, p38, c-Jun N-terminal kinase (JNK), and ERK5 (**Figure 8**). In the MAPK/ERK pathway, when the transmembrane receptors are activated, the cytoplasmic complexes of growth-factor-receptor bound protein (GRB) 2 and son of sevenless (SOS) are recruited to the inner surface of the cell membrane. From the RTK, the signal is sent to RAS. Then, with SOS’s assistance, RAS–GDP becomes RAS–GTP. Additionally, RAS-GTP functions as a molecular switch that sends signals downward, which causes downstream kinase RAF to be recruited and directly phosphorylated. RAF’s downstream kinase, MEK1/2, additionally catalyzes ERK1/2 (141, 142). The dual phosphorylation of MAP3Ks at the TGY motif is necessary for the activation of both JNK and P38. The most crucial MAP2Ks, MKK4 and MKK7, can be triggered to activate JNK (143). Activated JNK phosphorylates numerous cytoplasmic substrates, including cytoskeletal proteins and mitochondrial proteins like Bcl-2 and Bcl-xl, in addition to controlling a few transcription factors, including c-Jun, c-Fos, ATF-2, AP-1, p53, and Elk. The p38 goes from the cytosol to the nucleus after activation, where they activate downstream transcriptional targets such as PAX6, ETS1, PRAK, MK3, RARα, AP-1, ATF1, and CHO to control cellular processes (144) It is generally believed that overactivation of the MAPK/ERK signaling pathway is positively related to chemoresistance. According to one study, the calcium channel blockers lercanidipine and amlodipine could reverse chemoresistance and increase the doxorubicin sensitivity of GC cells through inhibiting the ERK/MAPK pathway (145). Chen et al. have discovered that after continuously stimulating with vincristine, MGC803 showed a high expression level of P-gp and developed resistance to vincristine. Following the addition of MEK inhibitor PD98059, P-gp level decreased significantly, and drug resistance was reversed (146). However, the JNK and P38 MAPK signaling pathways play a dual role in drug resistance. On the one hand, several chemotherapeutic drugs, such as cyclophosphamide and oxaliplatin, induce apoptosis that is reliant on P38 activation (147). Low et al. discovered that dual-specificity phosphatase 16 (DUSP16) increased drug resistance by preventing the activation of the P38 MAPK pathway and the JNK pathways, which led to reduced Bax accumulation in mitochondria to reduce apoptosis (148). However, under certain conditions, apoptosis resistance can also be mediated by the P38 MAPK pathway and the JNK pathway. According to one study, galectin-1 could promote tumor proliferation and medication resistance by activating the P38 MAPK pathway, which in turn increases the expression of Cox-2, which augments tumor angiogenesis and resistance to apoptosis (149). Prostate cancer cells become resistant to docetaxel-induced apoptosis when p38 is phosphorylated (150). HBV X protein can promote drug resistance and decrease adriamycin-mediated apoptosis by activating the JNK pathway (151).

According to Peng et al., sophoridine could mediate the MAPK signaling pathway, enhance the expression of estrogen-related receptor gamma (ESRRG) to promote β-catenin degradation, and inhibit the repair of double-stranded DNA breaks to caused cell cycle arrest at the G2/M phase, thereby lowering GC cell survival and increasing the efficiency of cisplatin. Because ESRRG was a downstream signaling protein of MAPK pathways, the activation of MAPKs ERK1/2, p38, and JNK1/2 promoted the phosphorylation of β-catenin (152). The tumor-suppressive properties of oridonin, an active compound derived from Rabdosia rubescens, had been demonstrated in various GC cell lines. Oridonin demonstrated the ability to inhibit cell proliferation by blocking cycle progression in the C2/M phase while also activating JNK signaling pathways to induce caspase-dependent apoptosis in the HGC27 cell line. However, JNK inhibitor SP600125 hindered the activation of JNK, leading to decreasing oridonin-mediated apoptosis (153). As a potential chemosensitizer, Hong et al. discovered that wogonin could promote oxaliplatin-induced apoptosis by activating phosphorylation of JNK signaling and raising nitrosative stress to accelerate excessive autophagy, thereby synergistically enhancing the chemotherapeutic impact of oxaliplatin on BGC832 cells *in vitro (* 154). As an active ingredient from Dioscorea zingiberensis C.H. Wright, it has been demonstrated that deltonin exhibited anti-cancer properties against a variety of cancer forms. Yang et al. discovered that deltonin might decrease the expressions of downstream apoptotic genes such Bad, Bid, and Fas by lowering the phosphorylation of P38-MAPK in GC cells. This inhibition was further strengthened when cisplatin was added, suggesting that deltonin may increase GC cells’ susceptibility to cisplatin treatment. Furthermore, by blocking the PI3K/Akt/mTOR signaling pathway, deltonin could also reduce the expression levels of important DNA-repair enzymes, such as Rad51 and murine double minute (MDM)2 (155). It had been demonstrated that ethanol extracted of Scutellaria barbata ESB increased the depolarization of the mitochondrial membrane and the activity of caspase-3 and caspase-9, which caused apoptosis. Additionally, the anti-tumor effect was greatly increased when cisplatin, etoposide, or doxorubicin were coupled with ESB; this may be connected to the MAPK signaling pathway. Furthermore, this impact could be lessened by MAPK inhibitors PD98059(an ERK1/2 inhibitor), SB203580(a p38 inhibitor), or SP600125 (a JNK inhibitor) (156). A scaffold protein called kinase suppressor of Ras 1 controls how the oncogenic mitogen-activated ERK/MAPK signaling cascade is initiated. In cisplatin-treated SGC7901 cells, etoposide-treated SGC7901 cells and cisplatin-resistant SGC7901 cells, Ginkgo biloba extract EGb could suppress proliferation and promote apoptosis by reducing the expression of KSR1, p-KSR1, ERK1/2, and p-ERK1/2. This suggested that EGb not only increased chemotherapy sensitivity but also reversed chemotherapy resistance (157).

### 4.4 P53 signaling pathway

**Figure 9:**
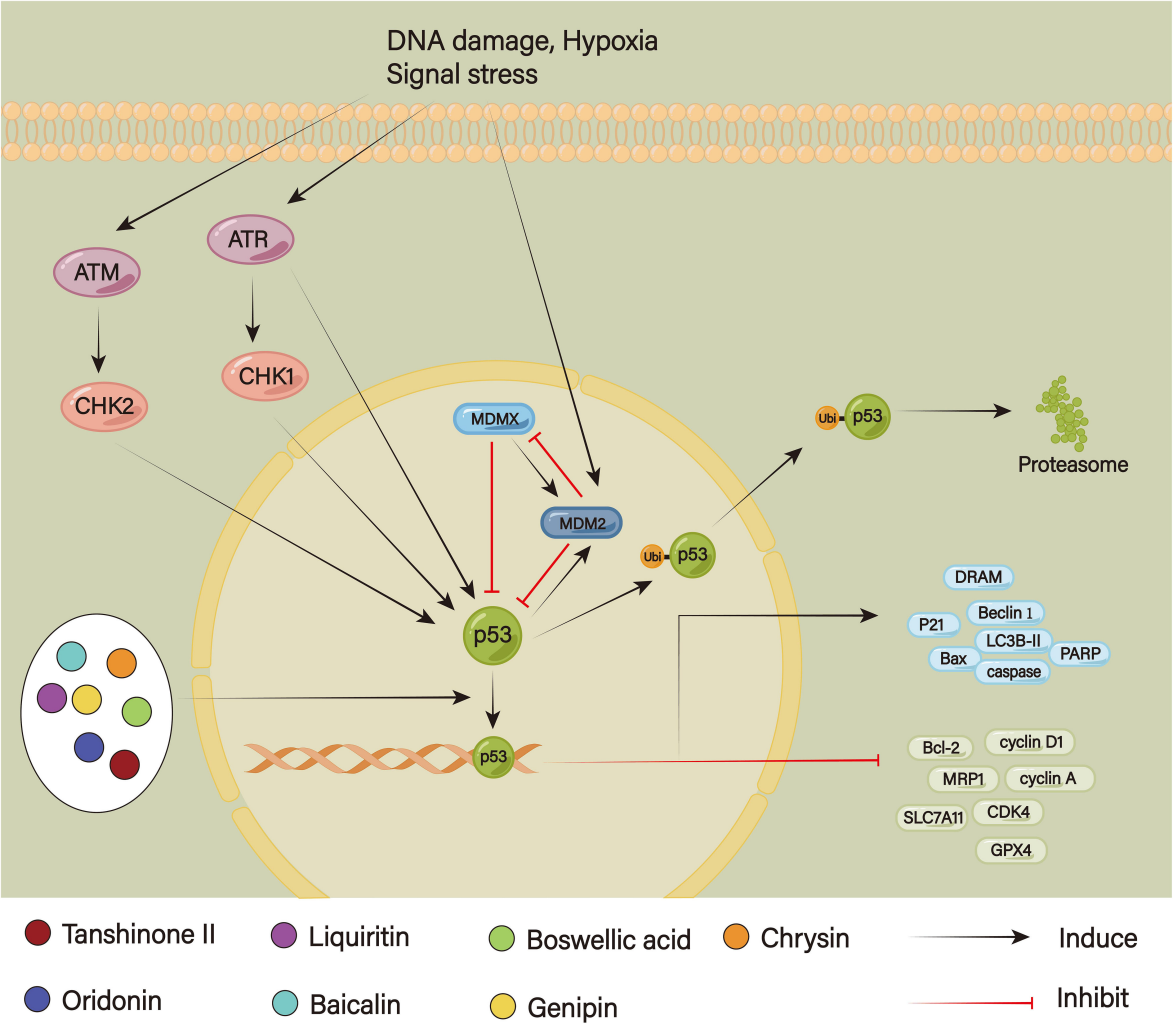
The p53 signaling pathway.

Unquestionably, p53 is a significant tumor suppressor that contributes to both normal proliferation and the inhibition of tumor growth (**Figure 9**). The p53 levels are regulated by negative feedback mediated by the E3 ubiquitin ligase MDM2 and its homolog MDMX (158). Signals of cell stress, including DNA damage and carcinogenic stress, cause p53 to become active. To lessen aberrant cell accumulation and stop tumors from forming, it, on the one hand, controls downstream signals, aids in the repair of DNA damage, stops the cell cycle, and transduces the caspase signal through the activation of the mitochondrial pathway or death receptor pathway to promote apoptosis when damage cannot be repaired (159). On the other hand, p53 ubiquitination by MDM2 and MDMX is followed by proteolytic hydrolysis or nuclear export to maintain the stability of the p53 level (160). Continuous stimulation can reduce P53 stability; additionally, mutant p53 is the most prevalent genetic abnormality in cancer cells, which are strongly linked to medication resistance. Di et al. have discovered that after continuous oxaliplatin stimulation, p53 ubiquitination was enhanced and its stability was damaged, thus inducing oxaliplatin resistance in CRC (161). Moreover, continuous temozolomide stimulation could cause P53 to become phosphorylated, which decreased drug absorption and improved DNA damage repair, leading to induced chemoresistance (162). According to Jing et al., miR-769-5p causes cisplatin resistance by promoting p53 degradation and blocking apoptosis via the ubiquitin-proteasome system (31). P53 can also simultaneously enhance cell survival and proliferation and control chemotherapy resistance through triggering various survival signaling pathways, including the NF-kB signaling pathway. Yang et al. have found that P53 could promote NF-kB p65 nuclear translocations in A549 or H358 cell lines, thereby enhancing the cell’s drug resistance to cisplatin and paclitaxel, which was significantly weakened after treatment with the NF-kB inhibitor PS1145 (163).

Xu et al. found that when doxorubicin and tanshinone II were administered together to doxorubicin-resistant SNU719 cells, the levels of p53 and Bax rose while those of Bcl-2 fell. However, doxorubicin alone had virtually no effect on the expression of genes linked to apoptosis. These suggested that via triggering the p53 signaling pathway, tanshinone II could induce apoptosis in doxorubicin-resistant SNU719 cells. Furthermore, it also enhanced the anticancer impact of doxorubicin via inhibition of MRP1 function (164). Because oridonin dramatically increased apoptotic cell and decreased cell viability, it remarkably amplified the anti-tumor impact of cisplatin. According to Bi et al., it increased the level of p53 expression by suppressing MDM2 expression through negative feedback regulation; at the same time, oridonin increased the pro-apoptotic function of p53 by suppressing the expression of anti-apoptotic genes Bcl-2 and up-regulating the expression of genes linked to pro-apoptosis, such as p53, p-p53, p21, and Bax (165). Liquiritin, a key component of licorice flavonoids, could increase cisplatin’s killing capacity and decrease resistance in SGC7901/DDP cells by preventing the cell cycle, triggering apoptosis and promoting autophagy. In the study, liquiritin and cisplatin caused cyclin D1 and cyclin A to all decrease at the same time, further arresting the G1/G0 cell cycle. Moreover, it increased LC3B-II and Beclin 1, which in turn stimulated caspase-8/-9/-3 and PARP cleavage, upregulating apoptosis autophagy (166). Scutellaria baicalensis is a Chinese herb that contains the potent compound baicalin. According to Shao et al., it dramatically rose p53 expression in HGC-27/OXA cells, which in turn targeted downstream ferroptosis activation by blocking SLC7A11 and glutathione peroxidase (GPX) 4 and promoting ROS accumulation, resulting in reverse oxaliplatin resistance (167). Extracted from frankincense, boswellic acid had been demonstrated to increase cisplatin-mediated apoptosis in GC cells by upregulating p53 expression and subsequently decreasing Akt downstream phosphorylation (168). A naturally occurring substance called Genipin, which comes from Gardenia jasminoides, may be a sensitizer to accelerate oxaliplatin-induced cell apoptosis and autophagy. It could trigger p53 expression, which in turn promoted the cleavage of PARP, caspase-9, and caspase-3 and rose damage-regulated autophagy modulator (DRAM) (169). According to Lee et al., Chrysin and 5-Fu worked together to enhance the anticancer effects of 5-Fu and overcame 5-Fu resistance *in vitro* by further upregulating p53 and subsequently stimulating p21 activity to block arrest in AGS/5-Fu cells (170).

### 4.5 Signal transducers and activators of transcription 3 signaling pathway

**Figure 10:**
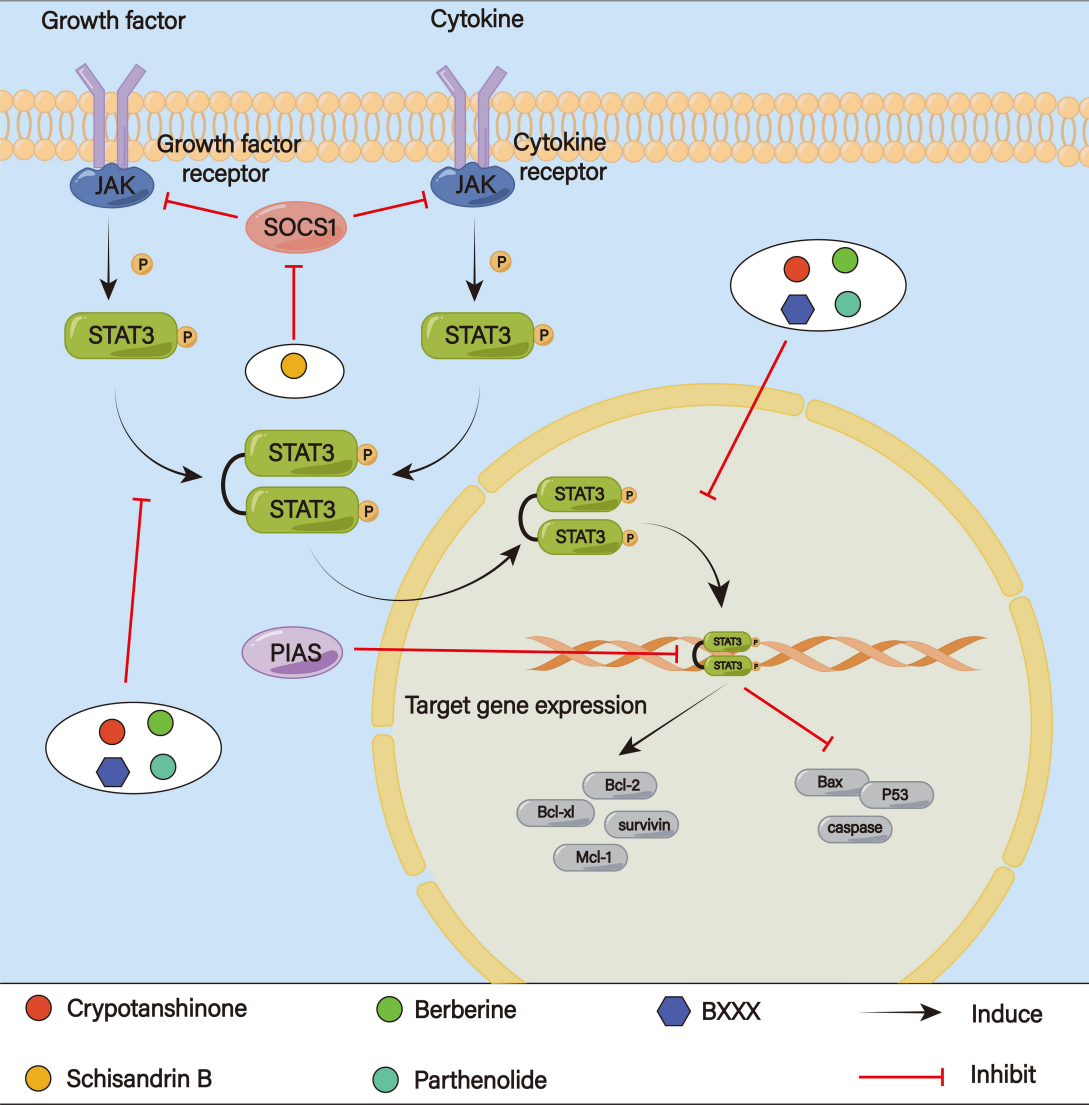
The STAT 3 signaling pathway.

Seven genes have been found by the STAT family; STAT3 in particular is generally thought to be linked to metastasis, cancer growth, and multidrug resistance (171) (**Figure 10**). The classical STAT3 signaling pathway is activated by a number of growth factors (EGF, FGF, IGF) and cytokines (IL-6, IL-10) binding to their appropriate reporters. The associated janus kinase (JAK) proteins are activated, self-phosphorylated, and transphosphorylate the receptor-associated tyrosine residues (172). Phosphorylated tyrosine residues are bound by STAT3 through its SH2 domain (173, 174). Homodimers are formed by the phosphorylated STAT3. Following its release from the receptors, importins quickly carry the pSTAT-pSTAT dimer into the nucleus. The dimer mediates subsequent biological functions, such as metastasis, cell death and drug resistance, by forming complexes with certain activators and binding to target gene promoters for transcription (175, 176). More and more studies have shown that the STAT3 signaling pathway plays a significant role in the regulation of tumor stemness and EMT, promoting EMT through key regulatory factors and subsequently producing cell stemness and chemoresistance (177). According to Shi et al., Glycochenodeoxycholic acid (GCDC) could reduce E-cadherin expression and enhance vimentin expression by activating the STAT3 pathway and then induce EMT and enhance the development of CSC-like characteristics in HCC cells, resulting in resistance to 5-Fu and cisplatin (178). By controlling metabolism, the STAT3 pathway can also affect cancer cell sensitivity to drugs. In prostate tumors, the activation of STAT3 signaling enhances glycolysis and proliferation in cancer cells, inhibits apoptosis, induces EMT mechanisms to facilitate cancer metastasis, and additionally activates drug resistance pathways (179). Wang et al. have found that the JAK/STAT3 pathway could control the expression of several genes involved in lipid metabolism, such as carnitine palmitoyltransferase 1B (CPT1B) and fatty acid β-oxidation (FAO), to mediate cancer stemness and chemoresistance (180). Chemoresistance could be reversed when FAO is blocked. JAK/STAT3 can also induce chemotherapy resistance in TME via inducing M2 polarization of macrophages (181).

Danshen’s fat-soluble diterpene, crypotanshinone, increased the effectiveness of 5-Fu in a mouse model of GC by reducing P-gp expression and altering the transcriptional activity of the MDR1 gene via the JAK2/STAT3 signaling pathway (182). Additionally, Cao et al. discovered that crypotanshinone reversed 5-Fu resistance and boosted the inhibitory effect of 5-Fu in SGC7901/5-Fu cells by blocking the JAK/STAT3 signaling pathway, which in turn lowed the levels of Mcl-1, Bcl-xl, and Bcl-2 expression while raising Bax expression (183). The anticancer activity of doxorubicin is further enhanced by crypotanshinone, which significantly suppressed constitutive and phosphorylation of STAT3 Tyr705 to inhibit STAT3 activity. This lowed the levels of proteins encoded by downstream target genes (Bcl-xL, Mcl-1, survivin) (184). Schisandrin B could enhance 5-Fu sensitivity in GC cells and cooperate to stop 5-Fu-induced cell death *in vitro* and *in vivo*, according to He and colleagues. SchisandrinB may be responsible for controlling STAT3 upstream proteins (SOCS, PIAS, PTPs) which in turn may cause autophagy triggered by STAT3 signaling activation (185). Berberine may be able to target STATs and surviving cells in drug-resistant GC cells, increasing the sensitivity of these cells to 5-Fu (186). Parthenolide-treated SGC7901/DDP cells showed decreased STAT3 activation, which led to apoptosis by raising the expression of Bax, P53, and cleaved caspase-9/-3 protein and lowering that of Bcl-2 and Bcl-x. Therefore, by blocking the STAT3 signaling pathway, parthenolide could reverse cisplatin resistance in GC (187). A decoction of banxia xiexin (BXXX) could lower the expression levels of DNA methyltransferases PD-L1 and O6-methylguanine-DNA methyltransferase (MGMT), which worked through the IL-6/JAK/STAT3 pathway, in GC cells resistant to cisplatin (188). This stops GC from proliferating while intensifying cisplatin’s inhibitory actions. , 

### 4.6 Other signaling pathway

As a multifunctional cytokine, TGF-β plays a role in immune response, apoptosis, differentiation, and cell proliferation. When the TGF ligand binds to the type II TGF receptor, classical TGF signaling begins. The type I TGF-β receptor is then recruited and phosphorylated, which phosphorylates the transcription factor Smad and starts downstream signal transduction, particularly EMT (189). Recent research has suggested that the TGF-β pathway may play a key role in cancer treatment resistance (190). Isolequiritigenin, which was another natural flavonoid from licorice, prevented GRP78-mediated stemness by suppressing the expression of transcriptional factors (SOX2, Nanog), linked to stemness, and cell surface indicators (CD24, CD44, LGR5). Additionally, it inhibited MMP-9 and IL-6, which prevented CAFs from activating to decrease drug resistance and ultimately boost chemosensitivity to 5-Fu, hence reducing TGF-B release by GC cells (191). By suppressing cancerous inhibitor of PP2A (CIP2A) expression in cells, polyphyllin I counteracted TGF-β1-mediated downregulation of E-cadherin and upregulation of vimentin, indicating that it may prevent EMT-promoted invasion and improve effectiveness when used in conjunction with chemotherapy (192).

Cellular resistance to oxidative damage is regulated by Nrf2, a transcription factor linked to oxidative stress. Nrf2 shields cancerous cells from chemotherapy that results in chemoresistance, as well as healthy cells from ROS-induced DNA damage (193, 194). Le et al. discovered that baicalein might increase the sensitivity of cisplatin in drug-resistant cells by lowering the levels of Nrf2 and Keap1 in SGC7901/DDP cells while also lowering the expression of MDR1. Furthermore, by blocking the Akt/mTOR pathway and upregulating the expression of LC3B and beclin 1, baicalin could cause drug-resistant cells to undergo autophagy and death (195). According to Huang et al., Yi-qi-hua-yu-jie-du (YQHYJD) decoction could reverse 5-Fu resistance and speed up apoptosis. This could partially restrict cell stemness by reducing MDR1 and MRP1 expression by blocking activity of the PI3K/Akt/Nrf2 pathway (196). Glutathione metabolism is frequently dysregulated in cisplatin-resistant GC. Further investigations by Huang et al. had demonstrated that YQHYJD could mitigate cisplatin resistance. The underlying mechanism may involve the inhibition of the phosphorylation cascade activity within the Akt/GSK3β pathway and modulation of Nrf2 expression, thereby reprogramming glutathione metabolism and promoting ferroptosis (197).

The Wnt/β-catenin pathway, sometimes referred to as the classical Wnt pathway, is typically highly conserved and is triggered by extracellular Wnt ligands interacting to membrane receptors (Frizzled, LRP5/6) autocrinely or paracrinely. After activation, the Wnt/β-catenin pathway causes β-catenin to become stable and move to the nucleus, where it uses T-cell factor/lymphoid enhancer-binding factor (TCF/LEF) transcription factors to activate and control downstream target genes, ultimately increasing the expression of genes related to cell migration, differentiation, proliferation, and survival (198, 199). Chemotherapy resistance has been linked to the Wnt/β-catenin signaling pathway in a number of cancers, including GC (200). Hosseini et al. found that cornus officinalis extract could induce apoptosis of GC cell lines and effectively inhibit cell proliferation *in vitro (* 201). Subsequent investigation revealed that loganin, the primary active component of cornus officinals, combined with 5-Fu inhibited the Wnt/β-catenin pathway and reduced the accumulation of β-catenin in the cytoplasm and nucleus, thereby downregulating downstream targets and key proteins to significantly inhibit stem-like properties and migration, suggesting that loganin could be an efficient sensitizer to boost 5-Fu’s anti-tumor effect (202). According to research by Hou et al., cardamonin may enhance the chemosensitivity of the BGC823/5-Fu cell line to 5-Fu by suppressing the expression of Wnt target genes (β-catenin, TCF5) and interfering with the β-catenin/TCF4 complex formation. Moreover, it might promote Rh123 accumulation to prevent P-gp overexpression. Additionally, cardamonin and 5-Fu considerably slowed the growth of tumors *in vivo (* 203). When combined with cisplatin, ginsenoside Rg3 could further decrease SGC7901/DDP cell migration, proliferation, and EMT while promoting cell death. In the meantime, cisplatin and Rg3 could inhibit the expression of associated proteins in the Wnt/β-catenin signaling pathway, suggesting that Rg3 could regulate cisplatin resistance (204).

## 5 Conclusion

Chemotherapy resistance is still an urgent problem in the treatment of malignant tumors. Intracellular signal pathways are involved in numerous biological processes and have also been demonstrated to be intimately linked to the development of drug resistance. Currently, blockers that target genes or signaling pathways linked to drug resistance have been discovered but are not utilized widely in clinical settings due to high cost and severe side effects. TCM has a lengthy history, a distinct theoretical framework, exceptional clinical effectiveness, and a significant role in the management of cancerous tumors. According to numerous fundamental tests and clinical data conducted in recent years, TCM can be employed as an auxiliary sensitizer of traditional chemotherapy drugs, effectively improve anti-cancer efficacy and reverse chemotherapy resistance. This review summarizes the primary mechanism of GC chemoresistacne and focuses on mechanisms of enhancing chemotherapy sensitivity and reversing drug resistance in TCM from the standpoint of the signaling pathways, which include PI3K/Akt, NF-kB, MAPK, P53, STAT3, TGF-β, Nrf2, and Wnt/b-catenin, thereby affecting various biological processes, such as cell cycle, cell proliferation, migration, apoptosis, autophagy, ferroptosis, TME, EMT, DNA damage repair, and cell stemness (**Table 1**). Whether TCM is taken alone or in conjunction with other anticancer medications, we think it has a lot of potential as an ongoing and alternative clinical treatment for cancer.

**Table 1:** The main signaling pathways of TCM to increase the sensitivity of chemical drugs.

Monomer/Formula	TCM	Possible mechanism	Drug
Quercetin	Hawthorn, Licorice, Knotweed	Increasing PTEN expression, blocking the PI3K/Akt signaling pathway, downregulating the expression level and activity of P-gp	Oxaliplatin
Celastrol	Celastrus, Triperygium	Inhibiting the PI3K/Akt signaling pathway, reducing the expression of P-gp, MRP1, and BCRP	Cisplatin
		Inhibiting the NF-κB signaling pathway	5-Fu
Dihydroartemisinin	Artemisia annua	Inhibiting the PI3K/Akt signaling pathway, activating caspase-8/9/3, downregulating P-gp expression	Cisplatin
Ginsenoside Rg3	Panax ginseng	Upregulating miR-429, regulating SOX2 and the PI3K/Akt signaling pathway	Cisplatin
		Inhibiting the Wnt/β-catenin signaling pathway	Cisplatin
Jaridon 6	Rabdosia rubescens	Inhibiting the PI3K/Akt signaling pathway, inhibiting SIRT1 enzyme	Paclitaxel
Baicalein	Scutellaria baicalensis	Promoting PTEN accumulation, inhibiting the PI3K/Akt signaling pathway, downregulating HIF-1α expression	5-Fu
		Decreasing the levels of Nrf2 and Keap1, decreasing the MDR1 expression	Cisplatin
Berberine	Evodia rutaecarpa Coptidis Rhizoma	Reducing the expression of MDR1 and MRP1, inhibiting the PI3K/Akt signaling pathway, activating caspase-9/3	Cisplatin
		Mediating the STAT3 signaling pathway	5-Fu
mBZYQ	/	Inhibiting the PI3K/Akt signaling pathway, activating T lymphocytes, inhibiting PD-L1 expression	5-Fu
JPYW	/	Inhibiting the PI3K/Akt signaling pathway, inhibiting the expression of MDR1 and MRP1, increasing Bcl-2 and caspace-3	5-Fu
Tanshinone I	Salvia miltiorrhiza Bunge	activating NF-κB/caspase-3 (8)/GSDME axis	Cisplatin
Parthenolide	Tanacetum balsamita	Inhibiting the NF-κB signaling pathway	Paclitaxel
		Blocking the STAT3 signaling pathway, increasing the expression of Bax, P53 and cleaved caspase-9/3 protein, decreasing the expression Bcl-2 and Bcl-x.	Cisplatin
Curcumol	Turmeric	Upregulating miR-7, inhibiting the NF-κB/SNAIL axis	Cisplatin
Wogonin	Scutellaria baicalensis	Inhibiting the NF-κB signaling pathway, modulating 5-FU metabolic enzymes	5-Fu
		Activating the JNK/MAPK signaling pathway, raising nitrosative stress	Oxaliplatin
Curcumin	Turmeric	Inhibiting the NF-κB signaling pathway, downregulating the expression of Bcl-2 and Bcl-xL	etoposide doxorubicin
Paeoniflorin	Paeonia lactiflora pall	Inhibiting the NF-κB signaling pathway	5-Fu
Sophoridine	Sophora alopecuroides. L	Mediating the MAPK signaling pathway, enhancing ESRRG expression, inhibiting DNA damage repair	Cisplatin
Deltonin	Dioscorea zingiberensis C.H. Wright	inhibiting the PI3K/Akt/mTOR signaling pathway, inhibiting the P38/MAPK signaling pathway, inhibiting DNA damage repair	Cisplatin
Scutellaria barbata ESB	Scutellaria barbata	Mediating the MAPK signaling pathway, activating caspase-3/9, depolarization of the mitochondrial membrane	Etoposide, Doxorubicin, Cisplatin
EGb	Ginkgo biloba	Inhibiting the KSR1-mediated ERK/MAPK signaling pathway	Cisplatin
Tanshinones II	Salvia miltiorrhiza Bunge	triggering the p53 signaling pathway, upregulating the expression of p53 and Bax, downregulating Bcl-2 expression	Doxorubicin
Oridonin	Rabdosia rubescens	increasing the level of p53 expression and Bax, downregulating Bcl-2 expression	Cisplatin
Liquiritin	Licorice	Increasing the expression of P53 and p21, decreasing cyclin D1, cyclin A and CDK4, activating caspase-8/9/3 and PARP, upregulating LC3B-II and Beclin 1	Cisplatin
Baicalin	Scutellaria baicalensis	Increasing P53 expression, blocking SLC7A11 and GPX4, promoting ROS accumulation	Oxaliplatin
Boswellic acid	Frankincense	upregulating p53 expression, decreasing Akt phosphorylation	Cisplatin
Genipin	Gardenia jasminoides	Increasing P53 expression, promoting the cleavage of PARP, caspase-9, and caspase-3 and DRAM	Oxaliplatin
Chrysin	Bignoniaceae plant oryx, propolis	Increasing P53 expression	5-Fu
Crypotanshinone	Salvia miltiorrhiza Bunge	Mediating the JAK/STAT3 signaling pathway, reducing the expression of P-gp and MDR1.	5-Fu
		Inhibiting the JAK/STAT3 signaling, increasing Bax expression, decreasing Mcl-1, Bcl-xl, and Bcl-2 expression	Doxorubicin
Schisandrin B	Schisandra chinensis	Mediating SOCS, PIAS and PTPs, activating the STAT3 signaling pathway	5-Fu
BXXX	/	Mediating the IL-6/JAK/STAT3 axis	Cisplatin
Isoliquiritigenin	Licorice	Inhibiting the expression of SOX2, Nanog, CD24, CD44, LGR5, MMP-9 and IL-6, reducing TGF-B release	5-Fu
Polyphyllin I	Polyphylla	Counteracting TGF-β1-mediated downregulation of E-cadherin and upregulation of vimentin	Cisplatin
YQHYJD	/	Blocking the PI3K/Akt/Nrf2 axis, decreasing the expression MDR1 and MRP1	5-Fu
		Blocking the Akt/GSK3β/Nrf2 axis, reprogramming glutathione metabolism	Cisplatin
Loganin	Cornus officinali	Inhibiting the Wnt/β-catenin signaling pathway, reducing the accumulation of β-catenin	5-Fu
cardamonin	Alpiniae katsumadai	Inhibiting the Wnt/β-catenin signaling pathway, breaking β-catenin/TCF4 complex formation	5-Fu

However, there are still certain shortcomings and restrictions in pertinent studies as of right now. Most of the existing studies focus on the role of a single signaling pathway or a single gene, and do not involve the interaction between pathways. In addition, the current research on the pharmacological mechanism of TCM mostly focuses on *in vitro* cell experiments, lacking a dearth of adequate and trustworthy *in vivo* experimental results as well as clinical efficacy data. TCM has the characteristics of multi-components and multi-targets, and the same monomer may affect multiple targets, which is incompatible with the concept of accurate targeted therapy in modern medicine. The use of the TCM formulas should be based on the treatment with syndrome differentiation, which is one of the features of TCM theoretical system; however, at the moment, nearly all research does not include the syndrome differentiation. Lastly, the extraction and clinical application of active components present additional challenges, including increasing drug extraction rate, increasing drug concentration, maintaining drug stability, improving bioavailability, and pharmacokinetics.

This review aims to present a new theoretical foundation for overcoming chemotherapy resistance in GC, as well as ideas for the development of new chemotherapy sensitizers and a favorable research direction, in order to provide a better, safer and more effective treatment plan and drug selection for enhancing the anti-tumor effect of traditional chemotherapy drugs and reversing chemotherapy resistance in the future.

## Glossary

GC: gastric cancer
HER2: human epidermal growth factor receptor 2
CPS: combined positive score
PD-L1: programmed cell death 1 ligand 1
FDA: food drug administration
TCM: traditional Chinese medicine
DHD4: DNA-binding protein 4
ABC: ATP-binding cassette
P-gp: P-glycoprotein
PI3K: phosphatidylinositol-3-kinases
Akt: protein kinase B
mTOR1/2: mammalian target of rapamycin complex 1/2
MRP1: multidrug resistance-associated protein 1
Bcl-2: B-cell lymphoma-2
MOMP: mitochondrial outer membrane permeabilization
BALF1: bamH1 A fragment leftward reading frame 1
TNF: tumor necrosis factor
NF κB: nuclear factor kappa-beta
ANXA1: annexin A1
HULC: highly up-regulated in liver cancer
FoxM1: forkhead box protein M1
ATG5: autophagy-related protein 5
ROS: reactive oxygen species
MDA: malondialdehyde
GSH: glutathione
Keap1: kelch-like ECH-associated protein 1
Nrf2: NF-E2-related factor 2
DDR: DNA damage response
NER: nucleotide excision repair
PARP1: Poly (ADP-Ribose) polymerase 1
BER: base excision repair
ERCC: excision repair cross-complementing gene
TME: tumor microenvironment
CAFs: cancer-associated fibroblasts
SDF-1: stromal cell-derived factor-1
TAMs: tumor-associated macrophages
MSCs: mesenchymal stem cells
MDR1: multi-drug resistance 1
EMT: epithelial-mesenchymal transition
TGF: transforming growth factor
Rab31: Ras-related protein 31
MUC-1: mucin 1
WTAP: wilms tumor 1 associated protein
ADAR1: adenosine deaminases acting on RNA1
AZIN1: antizyme inhibitor1
CSC: cancer stem cell
AKAP-L8: PRKA kinase anchor protein 8L
SCD1: stearoyl-CoA desaturase 1
MT1M: metallothionein 1 M
IGF2BP1: IGF2 mRNA binding protein 1
KHDRBS3: KH domain-containing RNA-binding signal transduction-associated protein 3
GLI1: glioma-associated oncogene homolog 1
SOX: SRY-box transcription factor
FUBP1: far upstream element-binding protein 1
MCM10: maintenance complex component 10
GRP75: glucose-regulated protein 75
NRP1: neuropilin 1
LDHA: lactate dehydrogenase A
ROR: regulator of reprogramming
HMGA2: high mobility group protein A2
TRIM14: tripartite motif 14
Dvl2: dishevelled 2
PIP3: phosphatidylinositol 3,4,5-trisphosphate
FOXO: forkhead box O
PTEN: phosphatase and tensin homolog deleted on chromosome 10
SIRT1: sirtuin1
KRAS: kirsten rat sarcoma virus oncogene homologue
DPD: dihydropyrimidine dehydrogenase
MAPK: mitogen-activated protein kinases
ERK: extracellular signal-regulated kinase
JNK: c-Jun N-terminal kinase
GRB: growth-factor-receptor bound protein
SOS: son of sevenless
FGFR: fibroblast growth factor receptor
ESRRG: estrogen-related receptor gamma
MDM: murine double minute
SLC7A11: solute carrier family 7 member 11
GPX: glutathione peroxidase
DRAM: damage-regulated autophagy modulator
STAT: signal transducers and activators of transcription
JAK: janus kinase
MGMT: O6-methylguanine-DNA methyltransferase
CIP2A: cancerous inhibitor of PP2A
TCF: T-cell factor
LEF: lymphoid enhancer-binding factor
